# Genetic variants of the DLK1, KISS1R, MKRN3 genes in girls with precocious puberty

**DOI:** 10.18699/vjgb-25-33

**Published:** 2025-04

**Authors:** E.A. Sazhenova, O.Yu. Vasilyeva, E.A. Fonova, M.B. Kankanam Pathiranage, A.Yu. Sambyalova, E.E. Khramova, L.V. Rychkova, S.A. Vasilyev, I.N. Lebedev

**Affiliations:** Research Institute of Medical Genetics, Tomsk National Research Medical Center of the Russian Academy of Sciences, Tomsk, Russia; Research Institute of Medical Genetics, Tomsk National Research Medical Center of the Russian Academy of Sciences, Tomsk, Russia; Research Institute of Medical Genetics, Tomsk National Research Medical Center of the Russian Academy of Sciences, Tomsk, Russia; Tomsk State University, Tomsk, Russia; Scientific Center for Family Health and Human Reproduction Problems, Irkutsk, Russia; Scientific Center for Family Health and Human Reproduction Problems, Irkutsk, Russia; Scientific Center for Family Health and Human Reproduction Problems, Irkutsk, Russia; Research Institute of Medical Genetics, Tomsk National Research Medical Center of the Russian Academy of Sciences, Tomsk, Russia; Research Institute of Medical Genetics, Tomsk National Research Medical Center of the Russian Academy of Sciences, Tomsk, Russia

**Keywords:** precocious puberty, hypothalamic-pituitary-gonadal axis, DLK1, KISS1, KISS1R, MKRN3 genes, преждевременное половое созревание, гипоталамо-гипофизарно-гонадная ось, гены DLK1,KISS1, KISS1R, MKRN3

## Abstract

Precocious puberty (PP, E30.1, Е22.8, Е30.9 according to ICD 10, MIM 176400, 615346) in children is a disorder in which secondary sexual characteristics appear earlier than the age norm. The timing of puberty is regulated by a complex interaction of genetic and epigenetic factors, as well as environmental and nutritional factors. This study aimed to search for pathogenic, likely pathogenic variants or variants of uncertain significance (VUS) in the KISS1, GPR54, DLK1, and MKRN3 genes in patients with the clinical picture of PP and normal karyotype by massive parallel sequencing. All identified genetic variants were confirmed by Sanger sequencing. The pathogenicity of identified genetic variants and the functional significance of the protein synthesized by them were analyzed according to recommendations for interpretation of NGS analysis results using online algorithms for pathogenicity prediction (Variant Effect Predictor, Franklin, Varsome, and PolyPhen2). Clinically significant genetic variants were detected in the heterozygous state in the KISS1R, DLK1, and MKRN3 genes in 5 of 52 probands (9.6 %) with PP, including 3 of 33 (9.1 %) in the group with central PP and 2 of 19 (10.5 %) in the group with gonadotropin-independent PP. Two children with gonadotropin-independent PP had VUS in the KISS1R gene (c.191T>C, p.Ile64Thr and c.233A>G, p.Asn78Ser), one of which was inherited from the father and the second, from the mother. The remaining patients with central PP had likely pathogenic genetic variants: DLK1:c.373delC(p.Gln125fs) de novo and DLK1:c.480delT(p.Gly161Alafs*49) of paternal origin. The third proband had a VUS variant in the MKRN3 gene (c.1487A>G, p.His496Arg), inherited from the father. All identified genetic variants were described for the first time in PP. Thus, in the present study, genetic variants in the KISS1R, DLK1, and MKRN3 genes in girls with PP were characterized.

## Abstract (ru)

Преждевременное половое созревание (ППС, Е30.1, Е22.8, Е30.9 по МКБ 10, MIM 176400, 615346) у детей – заболевание, при котором вторичные половые признаки появляются раньше возрастной нормы. Сроки полового созревания регулируются сложным взаимодействием генетических и эпигенетических факторов, а также факторов окружающей среды и питания. Цель настоящего исследования – поиск генетических причин формирования у девочек клинической картины ППС. Поиск клинически значимых генетических вариантов (патогенных, вероятно патогенных вариантов или вариантов с неопределенным клиническим значением (variant of uncertain significance, VUS)) проведен в генах KISS1, KISS1R (GPR54), DLK1 и MKRN3 у девочек с клинической картиной ППС и нормальным кариотипом методом таргетного массового параллельного секвенирования. Все найденные генетические варианты были подтверждены методом секвенирования ДНК по Сэнгеру. Патогенность идентифицированных генетических вариантов и функциональная значимость кодируемого ими белка про- анализированы с использованием онлайн-алгоритмов прогнозирования патогенности Variant Effect Predictor, Franklin и Varsome, а также PolyPhen2 (согласно рекомендациям по интерпретации результатов анализа NGS). Клинически значимые генетические варианты были обнаружены в гетерозиготном состоянии в генах KISS1R, DLK1 и MKRN3 у 5 из 52 пробандов (9.6 %) с ППС, из них 3 из 33 (9.1 %) – в группе с центральным ППС и 2 из 19 (10.5 %) – в группе с гонадотропин-независимой формой ППС. Два ребенка с гонадотропин-независимой формой ППС имели VUS в гене KISS1R (c.191T>C, p.Ile64Thr и c.233A>G, p.Asn78Ser), один из которых был унаследован от отца, второй – от матери. У остальных пациентов с центральным ППС были вероятно патогенные генетические варианты DLK1:c.373delC(p. Gln125fs) de novo и DLK1:c.480delT(p.Gly161Alafs*49) отцовского происхождения. Еще один пробанд имел вариант VUS в гене MKRN3 (c.1487A>G, p.His496Arg), унаследованный от отца. Все выявленные генетические варианты описаны впервые при ППС. Таким образом, в настоящем исследовании найдены новые генетические варианты в генах KISS1R, DLK1 и MKRN3 у девочек с преждевременным половым созреванием.

## Introduction

Precocious puberty (PP, E30.1, E22.8, E30.9 according to ICD 10, MIM 176400, 615346) is a disorder in which secondary sexual characteristics appear before the age of 8 in girls and before the age of 9 in boys, and, as a rule, there is an advancement of bone age by more than 2 years (Maione et al., 2021). The incidence of PP is 10–20 times higher in girls and varies widely across geographic regions, ranging from 0.217 to 26.28 per 10,000 girls and from 0.02 to 0.9 per 10,000 boys. The prevalence of familial cases of PP is 27.5 % (Brito et al., 2023).

PP can be gonadotropin-dependent (true, central), complete and incomplete, caused by premature reactivation of the hypothalamic- pituitary-gonadal (HPG) axis, and gonadotropinindependent (peripheral), developing as a result of excessive secretion of sex hormones by the gonads or adrenal glands, ovarian cysts, or human chorionic gonadotropin. The second form of PP is much less common, accounting for only 20 % of all PP (Shim et al., 2022). The causes of PP cannot be identified in most girls, therefore it is called idiopathic. If untreated, early puberty can lead to several serious complications, including short stature (caused by premature closure of the growth zones of tubular bones) and the formation of a dysplastic constitution (short limbs, elongated trunk, wide pelvis), psychological discomfort for girls and their parents. In girls, menstrual cycle disorders are observed, manifested in abnormal uterine bleeding, the development of polycystic ovary syndrome, premature ovarian failure, and, accordingly, early menopause. Earlier menarche in girls is also associated with an increased risk of breast cancer, endometrial cancer, obesity, type II diabetes, and cardiovascular diseases. PP can also be associated with organic brain lesions such as hypothalamic hamartoma, suprasellar arachnoid cysts, and hydrocephalus (Lagno et al., 2018; Peterkova et al., 2021).

Clinical features of PP include advanced growth spurt, progressive breast development in girls, and increased testicular volume in boys, and reflect high gonadotropin-releasing hormone (GnRH) levels and gonadotropin-stimulated sex steroid action (gonadarche). Accelerated growth velocity (>6 cm/year) and advanced bone age relative to biological age (>1 year or 2 SDS (standard deviation) points of chronological age) are common features of advanced PP. Hormonal findings supporting the diagnosis of PP include pubertal basal levels of luteinizing hormone (LH) or GnRH (Brito et al., 2023). The timing of puberty depends on genetic, epigenetic, and environmental factors. In recent years, genetic variants in the DLK1 (14q32), MKRN3 (15q11.2), KISS1 (1q32.1), and KISS1R (GPR54, 19p13.3) genes have been identified as hereditary causes of PP (Shim et al., 2022). However, in sporadic forms of PP, genetic variants in these genes are detected in only 10 % of cases (Canton et al., 2021, 2024). These genes primarily affect premature reactivation of the HPG axis and are directly involved in the formation of central PP. However, based on gene function, the clinical picture of peripheral PP may subsequently lead to central PP. For example, the presence of thelarche in girls after 2 years of age with a gonadotropinindependent form of PP increases the risk of subsequently developing central PP (Peterkova et al., 2021).

The KISS1 gene (MIM 603286) and its receptor KISS1R (MIM 604161) are responsible for the secretion of GnRH, participating in the regulation of endocrine function and the onset of puberty. The KISS1 gene encodes the kisspeptin protein, which stimulates the secretion of GnRH, and KISS1R is a regulator of this process and a key factor in the initiation of puberty, acting as a potent stimulator of the secretion of GnRH-dependent luteinizing hormone. It is expressed in various endocrine and gonadal tissues (Teles et al., 2008).

DLK1 (MIM 176290) is an imprinted gene expressed only on the paternal homologue and encodes an EGF-like growth factor. It is a membrane-binding protein that is involved in the Notch signaling pathway and promotes cell proliferation signals during neurogenesis. The product of this gene is also involved in osteogenesis, adipogenesis, hematopoiesis, and hepatocyte proliferation (Gomes et al., 2019; Macedo, Kaiser, 2019). In mice, Dlk1 is expressed prenatally in neuroendocrine tissues, including the pituitary gland, and postnatally in the hypothalamus, including the mediobasal hypothalamus, the control center for GnRH secretion (Shim et al., 2022). The product of this gene is also important for adipose tissue homeostasis. Genome-wide association studies have shown that paternally inherited single nucleotide variants in the DLK1 gene are associated with an earlier onset of menarche (Perry et al., 2014).

The imprinted and also paternally expressed MKRN3 gene (MIM 603856) encodes the macorine protein RING-finger 3, which belongs to the macorine family and is involved in controlling the onset of puberty by blocking the release of GnRH from the hypothalamus, thereby delaying the onset of puberty (Abreu et al., 2020). MKRN3 is responsible for protein ubiquitination, in which a ubiquitin moiety is attached to an intracellular protein to transfer it to the proteasome. Ubiquitination can also be an indicator of signal transmission for regulation of the cell cycle, differentiation, and morphogenesis (Abreu et al., 2020). Pathogenic and likely pathogenic variants in the MKRN3 gene are the most commonly known factors in genetic etiology of central PP, accounting for 19–33 % in familial and 2–3.9 % in sporadic cases (Valadares et al., 2019; Roberts, Kaiser, 2020)

This study aimed to identify clinically significant genetic variants in the KISS1, KISS1R (GPR54), DLK1, and MKRN3 genes in girls with a clinical picture of PP.

## Materials and methods

In the course of this study, a sample of 52 families (202 people in total) was formed based on the Scientific Center for Family Health and Human Reproduction Problems, Irkutsk. Each family consisted of a female proband with a clinical picture of PP, her parents and, in some cases, sisters and grandmothers. The study was conducted according to the provisions of the Helsinki Declaration of the World Medical Association. The study was approved by the Bioethics Committee of the Scientific Center for Family Health and Human Reproduction Problems (Protocol No. 1.1 dated 12.01.2023). Informed consent for participation in the study and DNA diagnostics was obtained from the patients’ parents. The clinical picture in the probands included PP with isosexual gonadotropin-dependent (ICD-10: E22.8, n = 33, age 7.4 ± 1.6 years) and gonadotropin- independent (ICD-10: E30.9, n = 19, age 6.9 ± 0.8 years) forms. Girls with organic lesions of the central nervous system were not included in the study.

Description of patient subgroups: • girls with the isosexual gonadotropin-dependent form of PP, under 8 years old, exhibiting accelerated physical development (height SDS +1 or more), with their sexual development corresponding to Tanner stages 2–4, levels of pituitary gonadotropic hormones corresponding to pubertal values, and a positive buserelin test. Additionally, they have enlarged mammary glands and uterus confirmed by ultrasound, and their biological age does not match their chronological (passport) age; • girls with the gonadotropin-independent form of PP, under 8 years old, with either accelerated or normal physical development (height SDS +1 or more), advanced sexual development corresponding to Tanner stage 2, levels of pituitary gonadotropic hormones corresponding to prepubertal values, and a negative buserelin test. Additionally, they exhibit enlarged mammary glands and uterus, which is confirmed by ultrasound.

All probands underwent standard cytogenetic analysis, which showed a normal karyotype in all cases. Karyotyping was performed using a research-grade microscope AxioImager (Carl Zeiss, Germany).

Genomic DNA was isolated from venous blood by phenolchloroform extraction. The concentration of the original samples was estimated using a Nanodrop 1000 spectrophotometer (ThermoFisher Scientific, USA). Genotyping of all exons in the KISS1, GPR54 (KISS1R), DLK1, and MKRN3 genes was performed using targeted massive parallel sequencing (NGS) of these genes using a MiSeq sequencer and a MicroKit (2x150) (Illumina, USA). For this purpose, amplification of long DNA fragments (Long-range PCR) was used. To obtain the nucleotide sequence, the UCSC In-Silico PCR genome browser was used, which contains information on genome sequences (hg38 assembly). The obtained nucleotide sequence was then used to select primers using the Primer-BLAST bioinformatics program provided by the National Center for Biotechnological Information (NCBI) (Table 1).

**Table 1.**
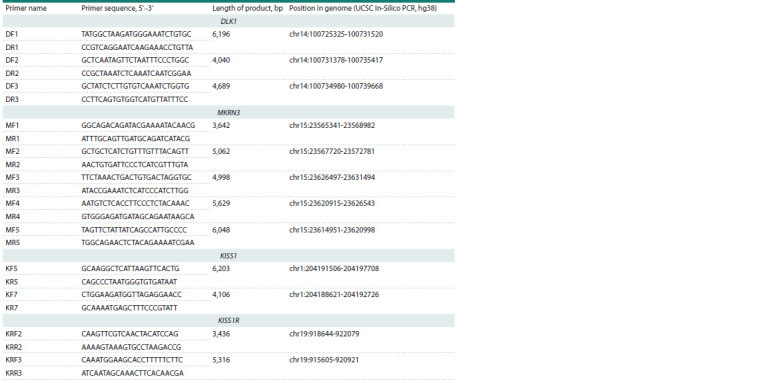
Sequences of the oligonucleotide primers used to generate libraries for targeted massive parallel sequencing of the KISS1, KISS1R, DLK1, and MKRN3 genes

Amplification of target fragments was performed using the BioMaster HS-Taq PCR (2x) kit (Biolabmix, Russia) according to the manufacturer’s protocol with the following PCR conditions: 95 °C for 5 min; 36 cycles: 95 °C for 40 s, 60 °C for 50 s, 68 °C for 1 min. The concentration of target fragments was determined using a Qubit 4.0 fluorimeter (ThermoFisher Scientific, USA). The reaction products were purified from impurities using a Sephadex G50 solution (Sigma, USA). The quality of reads was assessed using FastQC v0.11.8, after which trimming of the remaining adapter sequences and lowquality reads was performed using Trim-Galore

All detected genetic variants were confirmed using Sanger sequencing. The primer sequences are presented in Table 2. The pathogenicity of the identified genetic variants was analyzed using online pathogenicity prediction algorithms: Variant Effect Predictor (http://www.ensembl.org/Tools/ VEP), Provean (http://provean.jcvi.org/genomesubmit_2. php?species=human), Franclin (https://franklin.genoox. com/clinical-db/variant/snp/chr15-23621174-GC-G-hg38), VarSome (https://varsome.com/variant/hg19) and PolyPhen2 (http://genetics.bwh.harvard.edu/pph2/) according to the recommendations for interpreting the results of NGS analysis (Eijkelenboom et al., 2019; Ryzhkova et al., 2019). The following databases were used to determine the frequency of identified mutations in population samples in order to exclude polymorphic variants in patients: Exome Aggregation Consortium (http://exac.broadinstitute.org/), Exome Variant Server (http://evs.gs.washington.edu/EVS), 1000 Genomes Project (http://browser.1000genomes.org/index.html), which are recommended for interpreting data obtained using NGS (Eijkelenboom et al., 2019; Ryzhkova et al., 2019).

**Table 2.**
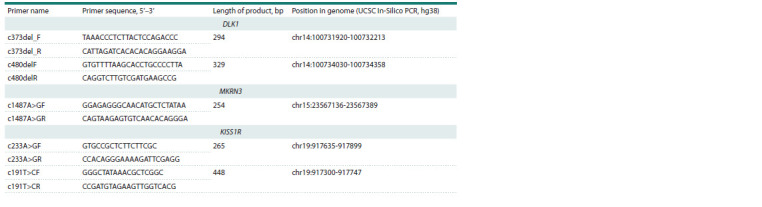
Sequences of oligonucleotide primers used for Sanger sequencing of the KISS1R, DLK1 and MKRN3 genes

The study was conducted using equipment from the Core Medical Genomics Facility of the Tomsk National Research Medical Center of the Russian Academy of Sciences, Tomsk.

## Results

Clinically significant genetic variants (likely pathogenic and variants of uncertain significance (VUS)) were identified in the KISS1R, DLK1 and MKRN3 genes in five of 52 probands (9.6 %) with PP, including three of 33 (9.1 %) in the group with central PP and two of 19 (10.5 %) in the group with gonadotropin-independent PP.

The main clinical characteristics of the phenotype of patients with the identified genetic variants are presented in Table 3. It is noteworthy that in three cases, the patients had obesity, which could contribute to the development of PP (Song et al., 2023).

**Table 3.**
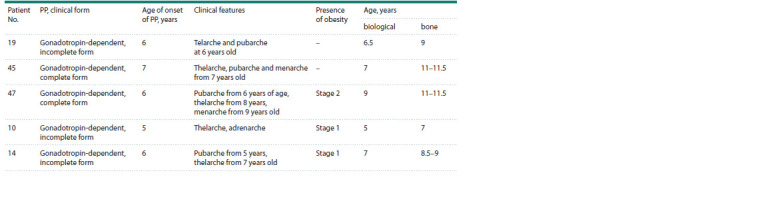
The main clinical characteristics of the phenotype of patients with the identified genetic variants

The clinically significant variants identified resulted in missense amino acid substitutions in three cases. Two variants were represented by single nucleotide deletions resulting in a reading frame shift. Table 4 and the Figure describe the spectrum of genetic variants identified in patients, which were registered in the heterozygous state in all cases, and also present their pedigrees.

**Table 4.**
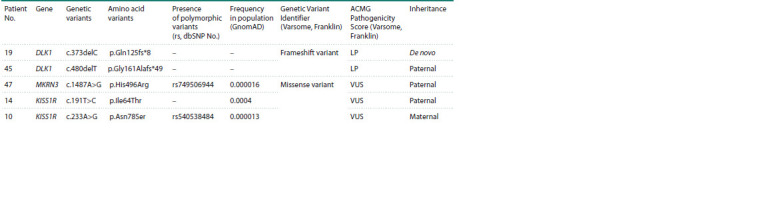
Position and characteristics of the identified genetic variants in patients with precocious puberty Notе. LP – likely pathogenic variant, VUS – variant of uncertain significance.

**Fig. 1.**
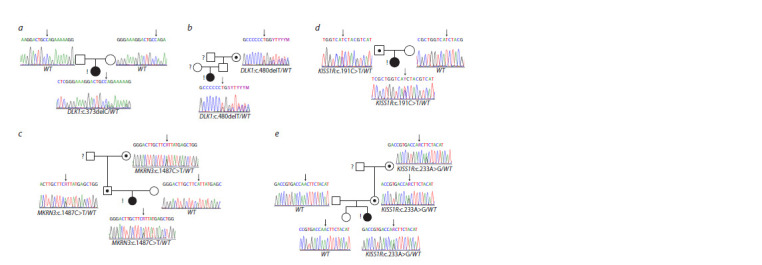
Pedigrees of patients with mutations in the KISS1R, DLK1, and MKRN3 genes available for familial segregation analysis. Pedigrees of patients: a – No. 19; b – No. 45; c – No. 47; d – No. 14; e – No. 10; squares represent male family members; circles represent female family members; black symbols represent clinically affected family members; white symbols represent clinically unaffected carriers; black dot represents clinically unaffected carriers with a detected genetic variant; question mark represents unknown phenotype; exclamation mark represents the proband in each family; WT represents the wild-type genotype status.

A total of five genetic variants located in the coding region of the studied genes were identified. Two likely pathogenic variants were identified in the DLK1 gene (c.373delC, p.Gln125fs and c.480delT p.Gly161Alafs*49) (Table 4, the Figure a, b). The first was located in the exon 4, and the second, in the exon 5. In both cases, these variants led to a reading frame shift and the formation of a stop codon and, as a consequence, to a shortening of the synthesized protein.

The DLK1 gene has five exons. The DLK1 protein structure consists of a transmembrane domain with six epidermal growth factor (EGF)-like repeats and a protease-sensitive sequence – target of tumor necrosis factor α-converting enzyme (TACE), a transmembrane domain, and a short cytoplasmic domain (Sánchez-Solana et al., 2011).

In the present study, both the first and second variants are located in the region containing EGF repeats, which are crucial for inhibiting the activity of Notch transmembrane proteins. These proteins act as transcriptional activators in complex with CSL family transcription factors (Baladrón et al., 2005; Gomes et al., 2019). The first variant is located in the third repeat, and the second, in the fourth repeat. Both genetic variants are described for the first time in PP. Previously, the c.479delC(p.Pro160fs*50) variant was described for this pathology (Gomes et al., 2019; Yuan et al., 2022); it is located near the DLK1:c.480delT(p.Gly161Alafs*49) variant described by us.

One missense variant of uncertain clinical significance (c.1487A>G, p.His496Arg, rs749506944) was found in the exon 4 of the MKRN3 gene. The frequency of this variant in the GnomAD database is extremely low (0.000016), and it is found only in the European population. The MKRN3 protein has four zinc finger domains: three RNA-binding C3H1 motifs and one protein-binding domain C3HC4, which is responsible for the activity of ubiquitin ligase. The MKRNTable specific Cys-His domain, which is part of the protein, has an unknown function. The genetic variant identified in this study is located in the region of RNA-binding C3H1motifs. Predictive programs prediction and the low frequency of this variant in the population indicate that this missense variant can be associated with the development of PP.

Genetic variants in the DLK1 and MKRN3 genes were inherited from fathers and paternal grandmothers in two cases, and de novo inheritance was observed in one family (No. 19) (Table 4, the Figure a–c). These genes are imprinted and are expressed only on the paternal chromosome. Fathers inherit this variant from their mothers, so this genetic variant is not active in fathers and there are no clinical manifestations of this disease. Indeed, fathers in families No. 45 and 47 did not have PP. At the same time, paternal grandmothers should have clinical signs of PP, since they have an active homologue. However, according to the survey, paternal grandmothers also did not have such disorders, which indicates incomplete penetrance of the identified genetic variants.

The other two genetic variants were found in the KISS1R gene, c.191T>C, p.Ile64Thr and c.233A>G, p.Asn78Ser; these were missense variants located in the first exon. In the first case, the proband inherited the variant from her father, who had no clinical manifestations of PP (the Figure d, e). In the second family, in addition to the proband, the mother and maternal grandmother had this genetic variant, and also had no cases of PP. The proband’s sister was not diagnosed with this disease. With regard to PP, all this indicates incomplete penetrance of the clinical phenotype.

The KISS1R gene has five exons. The protein coded by this gene, GPR54, is located in the cell membrane and has an extracellular N-terminal domain, followed by seven transmembrane helices with three intracellular and three extracellular loops, and ends with a C-terminal cytoplasmic domain. The variants identified in this gene are located in the first transmembrane helix.

In two families, unique genetic variants were found that were not repeated in unrelated patients (Table 4). In the remaining families, the genetic variants found in this study were observed in population samples with frequencies ranging from 0.000013 to 0.0004 (according to GnomAD). As can be seen, these variants are extremely rare in populations, which may indicate their pathogenic nature

Thus, in this study, it was shown that in the group of girls with PP in the KISS1R, DLK1 and MKRN3 genes, in 9.6 % (9.1 % in the group with central PP and 10.5 % in the group with gonadotropin-independent PP) of cases, likely pathogenic variants and variants with uncertain clinical significance (VUS) are present, which may be a potential cause of PP formation. All genetic variants found in this study are described in this disease for the first time. Identification of new genetic variants will allow a better understanding of the contribution of genetic causes to the development of PP.

## Discussion

Female reproductive process is a well-organized and tightly controlled system governed by the HPG axis. The main element of this axis is the pulsatile secretion of GnRH, which regulates the production of gonadotropins (follicle-stimulating hormone (FSH) and luteinizing hormone (LH)) by the anterior pituitary gland during puberty and maintains normal cycles in adults. GnRH and gonadotropin production are also regulated by negative feedback from estrogens secreted by developing ovarian follicles

In most cases, PP is associated with variants in the DLK1, MKRN3, KISS1, and KISS1R genes. Indeed, in our study, we found likely pathogenic variants and variants with uncertain clinical significance in the DLK1, MKRN3, and KISS1R genes in five of 52 probands (9.6 %) in a sample of girls with a clinical picture of PP. This finding aligns with literature reports on the frequency of detection of genetic variants in PP (Canton et al., 2021, 2024). No genetic variants were identified in the KISS1 gene in the sample of probands with a clinical picture of PP. This may be due to the limited number of patients in this study, as well as the low frequency of genetic disorders in this gene associated with PP. Indeed, only a few genetic variants in the KISS1 gene have been described in this pathology (Silveira et al., 2010; Rhie et al., 2014). All identified genetic variants were in the heterozygous state, which is consistent with the literature data on the autosomal dominant nature of inheritance of genetic variants of these genes in PP.

In the present study, we found genetic variants in the KISS1R gene in girls with gonadotropin-independent PP, in all cases accompanied by thelarche. Thelarche refers to the development of the mammary glands and is a response to estrogen synthesis. It has been determined that the KISS1 and KISS1R genes are expressed in various tissues, including the gonads, and are able to influence the level of these hormones either through temporary activation of the HPG axis or directly through stimulation of the gonads (Hu K. et al., 2018; Yarmolinskaya et al., 2016).

The low frequency (approximately 10 %) of detection of genetic variants in the DLK1, MKRN3, KISS1, and KISS1R genes, primarily in sporadic cases of PP, suggests that some other mechanisms or genes may also be involved in the formation of PP. Indeed, epimutations (changes in the methylation status of CpG dinucleotides) in the DLK1/MEG3:IG-DMR and MKRN3:TSS-DMR imprinting centers, which control the expression of the imprinted DLK1 and MKRN3 genes, may also be the cause of formation of the clinical picture of PP. In support of this, A.P.M. Canton et al. (2021) identified various genetic and epigenetic disruptions in 36 (18 %) of 197 unrelated patients with PP, among which: in 24 cases (67 %), genetic disruptions were found in the KISS1R, KISS1, MKRN3 and DLK1 genes; in 7 cases (19 %), CNVs were detected (3 patients had a de novo deletion of 7q11.23 (Williams– Beuren syndrome), 3 probands had an inherited deletion of Xp22.33 and one patient had a de novo duplication of 1p31.3); epigenetic abnormalities of imprinted centers of the DLK1 and MKRN3 genes accounted for 3 cases (9 %); identification of the genetic variants of genes using whole exome sequencing revealed rare de novo variants of loss of gene function in a dominant state in two probands (5 %) such as pathogenic deletion with a reading frameshift in the TNRC6B gene (p.Gly665Leufs*35) and a likely pathogenic variant of a reading frameshift in the AREL1 gene (p.Ser229Phefs*3).

The TNRC6B gene (trinucleotide repeat containing adaptor 6B, region 22q13.1, OMIM 610740) encodes a protein with RNA-binding activity, which is involved in the regulation of gene expression. This gene plays a role in RNA-mediated gene silencing by both micro-RNAs (miRNAs) and short interfering RNAs (siRNAs). The AREL1 gene (apoptosis-resistant E3 ubiquitin protein ligase 1, region 14q24.3, OMIM 615380) encodes a protein that activates ubiquitin protein transferases, is involved in the negative regulation of apoptosis, protein ubiquitination, and is located in the cytosol

Meta-analysis of association studies has also expanded the range of genes that could potentially cause the development of PP. These include genes such as LIN28B and PROKR2, although their roles in this process are not so obvious (Perry et al., 2009).

The LIN28B gene (6q16.3, OMIM 611044) encodes a highly conserved RNA-binding protein that blocks LET7 family microRNAs and helps maintain the pluripotent state of embryonic stem cells by preventing differentiation, and is involved in metabolism and oncogenesis. It may also play a role in pubertal development. Several studies have shown that LIN28B is involved in forming the clinical presentation of PP, in particular, earlier development of thelarche, menarche, and pubarche (Ong et al., 2009; Perry et al., 2009; Hu Z. et al., 2016). However, another study assessed the association between LIN28B variants in 178 Brazilian children with PP, but did not find a causal relationship (Silveira-Neto et al., 2012). Moreover, genetic variants in LIN28B such as rs314276 have been reported to be associated with obesity, which is closely linked with PP (Ong et al., 2011). Thus, the role of the LIN28B gene in forming the clinical presentation of PP remains to be determined.

The PROKR2 gene (prokineticin receptor 2, 20p12.3, OMIM 607123) is a G protein-coupled receptor that is involved in the development of GnRH neurons, but neither developing nor mature GnRH neurons express prokineticin receptors. M. Fukami et al. (2017) reported that a PROKR2 variant is associated with the formation of central PP. In this case, a girl presented with thelarche at the age of 3 years and 5 months with blood gonadotropin and estradiol (E2) levels consistent with puberty. Molecular analysis revealed a heterozygous deletion c.724_727delTGCT in this gene, resulting in transcription terminations. This variant was also detected in the patient’s mother, who did not have PP. It has been shown that in the heterozygous state, this variant forms a heterodimer with the wild type, which acts as a gain-of-function variant leading to PP. Moreover, S. Sposini et al. (2015) demonstrated that in the absence of the 6th and 7th transmembrane domains in the PROKR2 gene, ligand-dependent signal transduction is enhanced. Thus, only certain variants in the PROKR2 gene in the heterozygous state can lead to the development of PP.

## Conclusion

The onset of puberty is controlled by the interaction between genetic, epigenetic and non-hereditary factors. PP is a result of premature activation of these interactions. In the present study, it was shown that in the group of girls with PP, 9.6 % (9.1 % in the group with central PP and 10.5 % in the group with gonadotropin-independent PP) of cases had likely pathogenic variants and variants with uncertain clinical significance in the KISS1R, DLK1 and MKRN3 genes, which can be a potential cause of PP. All genetic variants detected in this study are described in PP for the first time. Analysis of familial segregation showed that in all cases, the probands had genetically significant variants in the heterozygous state, which confirms the autosomal dominant nature of inheritance. In all cases where family material was available, only the probands exhibited the clinical picture of PP, indicating incomplete penetrance of the disease

Identification of genetic variants is necessary not only for molecular genetic confirmation of the diagnosis, but also for choosing the right tactics for patient management and medical genetic counseling of the family. A comprehensive and stepby- step study of genetic, epigenetic and non-hereditary factors can improve our understanding of the exact mechanism of PP.

## Conflict of interest

The authors declare no conflict of interest.
